# Multi-Modal versus Uni-Modal Treatment for the Recovery of Lower Limb Motor Function in Patients after Stroke: A Systematic Review with Meta-Analysis

**DOI:** 10.3390/healthcare12020189

**Published:** 2024-01-12

**Authors:** Alex Lando, Luisa Cacciante, Alessio Mantineo, Francesca Baldan, Paolo Pillastrini, Andrea Turolla, Giorgia Pregnolato

**Affiliations:** 1 Rehabilitation Unit, Department of Neuroscience, General Hospital—University of Padova, 35128 Padova, Italy; alex.lando@aopd.veneto.it; 2 Laboratory of Healthcare Innovation Technology, IRCCS San Camillo Hospital, 30126 Venice, Italy; alessio.mantineo@hsancamillo.it (A.M.); giorgia.pregnolato@hsancamillo.it (G.P.); 3 IRCCS San Camillo Hospital, 30126 Venice, Italy; francescabaldan3@gmail.com; 4 Department of Biomedical and Neuromotor Sciences, Alma Mater University of Bologna, 40128 Bologna, Italy; paolo.pillastrini@unibo.it (P.P.); andrea.turolla@unibo.it (A.T.); 5 Unit of Occupational Medicine, IRCCS Azienda Ospedaliero-Universitaria di Bologna, 40138 Bologna, Italy

**Keywords:** stroke, lower limb, rehabilitation, multimodal treatment, unimodal treatment, resistance, endurance, combined training

## Abstract

The aim of this study is to evaluate whether the multimodal treatment based on both resistance and endurance training for the recovery of lower limb function in post-stroke patients is more effective than unimodal treatment. Six electronic databases were searched. The included articles were firstly analysed for methodological quality and then quantitatively analysed for the following outcomes: endurance, knee-extensor muscle strength, gait speed, and aerobic capacity. The treatment effect was analysed with the mean difference (MD) or standardised mean difference (SMD). From a total of 4439 records, 10 studies met the inclusion criteria for the qualitative analysis, whereas 7 studies were included in the quantitative analysis. There is a significant difference favourable to the group with multimodal treatment for knee-extensor muscle strength (SMD = 1.25; 95% CI 0.97, 1.53, I^2^ = 42%), both for the affected and the unaffected side. Multimodal treatments are a valid choice in the field of post-stroke rehabilitation. In particular, the combination of resistance and endurance training is useful to maximise the recovery of knee-extensor muscle strength, which in turn could be beneficial for achieving upright position and walking, allowing patients to improve independence levels in their activities of daily life.

## 1. Introduction

According to the most recent Global Burden of Disease (GBD) 2019 estimates, stroke continues to be the second leading cause of death and the third leading cause of death and disability combined (measured by disability-adjusted life-years lost—DALYs) worldwide [1,2]. Advances in stroke acute treatment have led to more patients surviving the initial injury with varying degrees of disability [3]. Motor impairment, which can be defined as a loss or limitation of function in muscle control, movement, or mobility, is one of the most recognised impairments caused by stroke [4]. Usually, stroke survivors experience long-term difficulties in carrying out common activities of daily living (ADL) and have limited participation in social life [5,6] because of motor sequelae. In particular, approximately 30% of patients with a stroke have persistent difficulties in independent ambulation [7] and in other activities related to lower limb functioning (e.g., standing up, going up/downstairs). Indeed, as stated in the International Classification of Function, Disability, and Health Framework (ICF), stroke patients have to be considered not only for their clinical dysfunction but also as individuals embedded in a wider framework that encompasses activities and social participation [8]. Following this framework, improving motor functions could have a positive impact on the activities the individuals can perform, which in turn could help reintroduce the patients within their social and work contexts. Therefore, lower limb rehabilitation for restoration of gait and gait-related activities is considered one of the primary goals and a major priority in managing stroke patients [9].

Rehabilitation training is the most effective approach to reducing motor impairments after stroke [10] and must be focused on those outcomes that can influence positively the independence of ADLs. The improvement in global body functions (e.g., strength, balance, endurance, and aerobic capacity) is highly related to the improvement in lower limb functioning, and thus independence in ADLs [11].

Nowadays, many types of treatments can be provided for the functional restoration of the lower limb. Research studies in the neurorehabilitation field have demonstrated the effectiveness of several single-targeted interventions. Current data indicates that resistance training may be beneficial in supporting the recovery of stroke patients [12,13], and even endurance training is increasingly recognised as an important component of stroke rehabilitation [14]. Furthermore, there is a growing body of literature exploring the effect of multimodal treatments. Multimodal treatments refer to those treatments that use exercises to train different types of outcomes in the same session. An example of a multimodal treatment is one that trains both muscular strength and endurance. This specific type of training, which combines aerobic and resistance training modalities into a single time-efficient exercise session, is already supported by evidence regarding a healthy population and is highly recommended for individuals to meet current physical activity recommendations [15].

The guidelines for stroke rehabilitation in adults recommend specific training parameters to improve mobility. These include activity-specific and functional task practice, progressively more difficult and challenging [16]. Multimodal treatment incorporates many of these elements since it challenges patients’ abilities and improves their motor skills.

However, there is still not a firm conclusion on the effectiveness of multimodal treatment when compared to unimodal treatment. Therefore, there is a need to synthesise systematic knowledge from the literature, with the aim of reaching evidence-based conclusions on the effectiveness of multimodal treatments after stroke. Thus, given the lack of comprehensive systematic reviews on multimodal treatments for stroke survivors, in this study, we will focus on evaluating whether multimodal treatment, specifically based on resistance and endurance training for the recovery of lower limb motor function, is more effective than unimodal treatment after stroke.

## 2. Materials and Methods

The study design was a systematic review with meta-analysis and was conducted according to the Preferred Reporting Items for Systematic Reviews and Meta-Analyses (PRISMA) statement [17]. The protocol was registered a priori in the PROSPERO database under the following registration number: CRD42022313023.

### 2.1. Data Sources and Searches

Publications were searched in Pubmed, Cochrane, PEDro, Embase, Scopus and Web of Science databases. The last search was launched on 10 February 2022. A detailed description of the search strategy is presented in Supplementary Materials A.

### 2.2. Study Selection

We included randomised controlled trials (RCTs) that enrolled adult participants with a diagnosis of stroke and undergoing an intervention defined as a multimodal treatment (e.g., combined lower limb resistance training and endurance training, with or without technological devices), as compared with a unimodal treatment or conventional treatment or no treatment, for the recovery of lower limb functions and aerobic functions (i.e., endurance, knee-extensor muscle strength, gait speed, and aerobic capacity). Only studies written in English were included. No date restriction was applied. The grey literature was not searched in this review. The study selection process consisted of two steps of screening using Rayyan QRCI online software [18]: (a) title and abstract screening and (b) full-text screening. For both steps, two pairs of blind independent reviewers (AL, AM, FB, GP) screened the articles, and then a third author (LC) resolved any conflicts. At the end of the screening process, the same procedures were used for the assessment of the methodological quality.

### 2.3. Outcomes

The primary outcome was an improvement in endurance (measured with the six-minute walking test—6MWT) in patients undergoing multimodal treatments versus unimodal treatments, conventional treatments, or no treatment. The secondary outcomes were knee-extensor muscle strength (measured with one maximal repetition), gait speed (measured with the ten-metre walking test—10MWT) and aerobic capacity (measured with the VO_2_ peak).

### 2.4. Data Extraction and Management

A data extraction form was filled in by two independent reviewers (AL, AM) with all the following relevant data: first author, year of publication, group characteristics, number of participants in each group, interventions, multimodal treatment description, outcome measures, and conclusions drawn by the authors.

### 2.5. Assessment of Risk of Bias in Included Studies

The included studies underwent a methodological quality assessment for the risk of bias using the revised Cochrane risk-of-bias tool for randomised trials (RoB2) [19]. The risk of bias was assessed at the individual outcome level only for the primary outcome (i.e., endurance). We evaluated the following domains: (1) bias arising from the randomisation process; (2) bias due to deviations from intended interventions; (3) bias due to missing outcome data; (4) bias in the measurement of the outcome; and (5) bias in the selection of the reported result. For each domain, the judgement on the risk of bias was expressed as “low”, “high”, or “some concern”.

### 2.6. Measures of the Treatment Effect

For statistical analysis, we used Review Manager 5.4.1. We evaluated treatment effects using mean difference (MD) or standardised mean difference (SMD) in the cases that the same outcome measures were extracted from studies or not, respectively. The confidence interval (CI) for continuous outcomes was set at 95%.

### 2.7. Assessment of Heterogeneity

We assessed heterogeneity with the I^2^ statistic, setting the cut-off value at 50% and considering interventions and outcome measures.

### 2.8. Data Synthesis

We conducted a meta-analysis using either a random-effects model or a fixed model, depending on the heterogeneity of the results, with 95% CI using RevMan 5.4.1.

### 2.9. Subgroup Analysis

We planned a subgroup analysis regarding lower limb muscle strength based on the body side affected by stroke (i.e., affected or unaffected side).

## 3. Results

**Figure 1:**
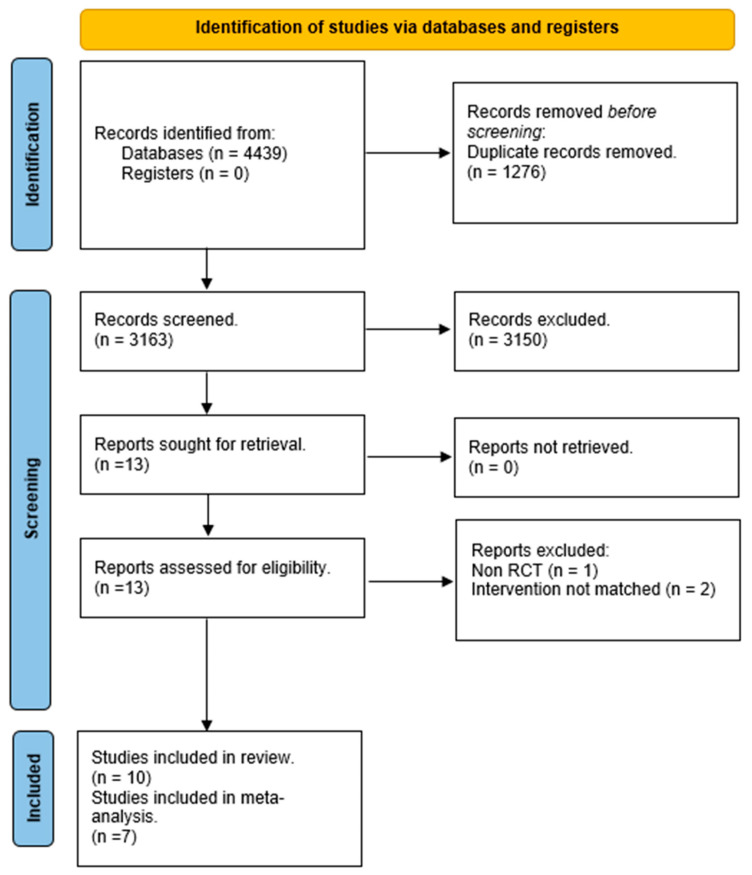
PRISMA flow diagram.

Our search identified 4439 results from six electronic databases. After removing 1276 duplicates, 3163 abstracts were screened. We excluded 3150 records due to unrelated target topics and then assessed 13 full-text articles for eligibility. After full-text screening, 10 studies met the inclusion criteria for qualitative analysis. At the end of the process, seven studies remained for quantitative analysis. The PRISMA flowchart of the review process is shown in Figure 1.

### 3.1. Included Studies

All the included studies were RCTs focused on rehabilitation with a multimodal approach for patients after stroke. Except for one study [20], which included patients with an average time from stroke of less than 3 months, all the other studies included patients with a time from stroke onset longer than 3 months. The overall number of participants included was 480, with 266 patients enrolled in the experimental groups and 214 patients in the control groups.

The dose and type of experimental treatment varied between the included studies. The duration of the training ranged from a total of 5 days [21] to 24 weeks [22], with a session frequency ranging from a minimum of 3 days-per-week [23,24,25,26] to 5 days-per-week [21,22,27,28].

In all the studies, the therapy was provided both with technology (e.g., biofeedback operating systems, leg press devices, treadmills, and cycle ergometers) and without technology (e.g., muscle contractions, elastic bands, speed walking, stepping). The most used tool among the studies was cycle ergometer from different manufacturers (e.g., MOTOMed VIVA Cycle and MOTOMed Viva2, Reck GmbH, Bremen, Germany; K400, Keiser SportsHealth, Inc., Fresno, CA, USA; Ergoline, Ergoselect 1000, Blitz, Germany; Ergoselect 200P, Blitz, Germany).

For the control groups, the treatments provided to patients ranged from no treatment [25,26] to conventional treatment [20,21,28,29], the same training sessions with lower intensity than the experimental group, or sham training [22,23,24,27].

With regard to outcomes, endurance was assessed in seven studies [20,21,22,23,24,27,29], lower limb strength for both the affected and the unaffected side in six studies [21,22,23,24,26,27], gait speed in five studies [20,21,24,25,26], and t aerobic capacity in three studies [22,24,27].

More details on the characteristics of the included studies are presented in Table 1.

### 3.2. Excluded Studies

After full-text screening, we excluded a total of three studies. One study [30] was not eligible as non-RCT, whereas the other two studies [31,32] did not evaluate a multimodal treatment, as described before.

Regarding quantitative analysis, three studies were excluded: two studies [20,23] did not report data as the mean and standard deviation, and one study [26] did not report the control group data.

### 3.3. Risk of Bias in the Included Studies

- Bias arising from the randomisation process: Six studies were assessed with a low risk of bias, as the authors described a correct randomisation process and, therefore, there were no differences between intervention groups related to this process. One study [20] was judged with a high risk of bias, as the participants were randomised according to clinical needs. Three studies [22,26,27] were judged with some concerns regarding the risk of bias, as no information was provided.
- Bias due to deviations from the intended interventions: Eight studies had a low risk of bias in this domain. Moreover, one study [25] had a high risk of bias because the participants, carers, and therapists were aware of the intervention received, and the drop-out rate was high (13%). Finally, one study [20] did not provide information, resulting in some concerns about the risk of bias.
- Bias due to missing outcome data: All the studies had a low risk of bias in this domain except for two studies, Refs. [20,25], which had a high risk of bias because several patients dropped out and no evidence was provided on missing data.
- Bias in measurement of the outcome: Five studies had a low risk of bias in this domain, whereas four studies [20,22,23,26] had a high risk of bias because the outcome assessor was not blinded or some outcome measures were collected only in the intervention group. One study [21] had some concerns about the risk of bias since the health professionals had free access to the subjects, making it difficult to guarantee the complete blinding of the evaluators.
- Bias in the selection of the reported result: One study [24] had a low risk of bias since the data were in accordance with the pre-registered study protocol. Another study [11] had a high risk of bias because the reported results were not in accordance with the study protocol, whereas for the other eight studies, there were some concerns about the presence of risk of bias since no information about the study protocol was provided.
- Overall bias: Two studies [24,29] had a low risk of bias, and for two other studies [27,28], there were some concerns about the judgement of the risk of bias. The remaining six studies had a high risk of bias.

**Figure 2:**
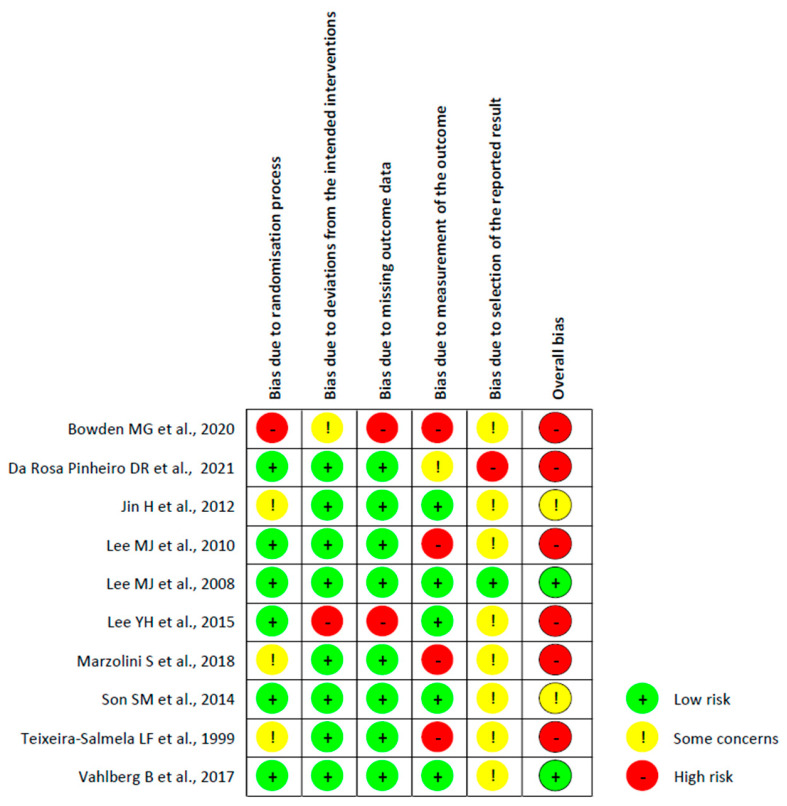
Risk of bias of the included RCTs using the ROB2 tool [20,21,22,23,24,25,26,27,28,29].

Figure 2 shows the risk of bias in the included studies, whereas a detailed description of the risk of bias assessment is included in Supplementary Materials B.

### 3.4. Effects of Intervention

#### 3.4.1. Effect of Multimodal Treatment on Endurance Compared to Unimodal and Usual Care Treatment

**Figure 3:**
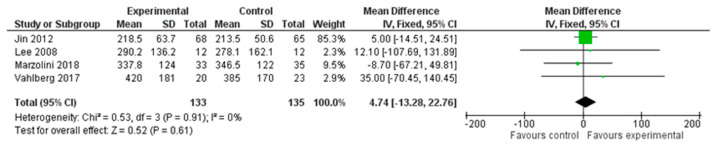
Comparison 1. Multimodal treatment versus unimodal or usual care treatment. Outcome: endurance [22,24,27,29].

A total of four studies, with an overall number of 268 participants, were analysed for endurance, measured with the six-minute walking test (6MWT). The analysis was performed using the mean difference (MD) with a fixed effect model and confidence interval (CI) of 95%. The meta-analysis did not show a significant difference between the two treatment modalities (MD = 4.74; 95% CI −13.28, 22.76, I^2^ = 0%). (Figure 3)

#### 3.4.2. Effect of Multimodal Treatment on Knee-Extensor Muscle Strength Compared to Unimodal Treatment

**Figure 4:**
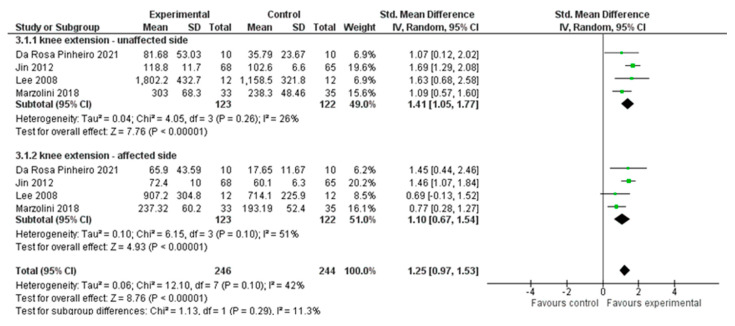
Comparison 2. Multimodal versus unimodal treatment. Outcome: knee-extensor muscle strength [21,22,24,27].

Four studies, with an overall number of 490 participants, were analysed to evaluate improvement in knee-extensor muscle strength. We performed a subgroup analysis based on the side affected (i.e., affected side or unaffected side) using the standardised mean difference (SMD) with a random effect model since all the included studies used different outcome measures for the strength assessment. The data showed significant differences in support for the experimental group (SMD = 1.25; 95% CI 0.97, 1.53, I^2^ = 42%), for both the affected muscles (SMD = 1.10; 95% CI 0.67, 1.56, I^2^ = 51%) and the unaffected side muscles (SMD = 1.41; 95% CI 1.05, 1.77, I^2^ = 26%). (Figure 4)

#### 3.4.3. Effect of Multimodal Treatment on Gait Speed Compared to Unimodal and No Treatment

**Figure 5:**
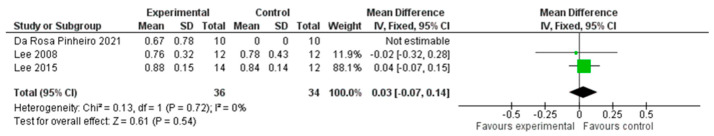
Comparison 3. Multimodal versus unimodal or no treatment. Outcome: gait speed [21,24,25].

We analysed three studies, including 70 participants overall, evaluating gait speed using the ten-metre walking test (10MWT). We performed the analysis using the mean difference (MD) with a fixed effect model and a confidence interval (CI) of 95%. The meta-analysis did not show a significant difference between the two treatment modalities (MD = 0.03; 95% CI −0.07, 0.14, I^2^ = 0%) (Figure 5).

#### 3.4.4. Effect of Multimodal Treatment on Aerobic Capacity Compared to Unimodal Treatment

**Figure 6:**
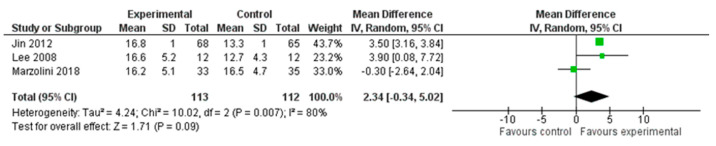
Comparison 4. Multimodal versus unimodal treatment. Outcome: aerobic capacity [22,24,27].

A total of three studies, with an overall number of 225 participants, were analysed for aerobic capacity, measured with the VO_2_ peak. We performed the analysis using the mean difference (MD) with a random effect model and a confidence interval (CI) of 95%. The meta-analysis did not show a significant difference between the two treatment modalities (MD = 2.34; 95% CI −0.34, 5.02, I^2^ = 80%). (Figure 6).

## 4. Discussion

The aim of this systematic review was to evaluate whether the multimodal treatment based on both resistance and endurance training for the recovery of lower limb function in patients after stroke, is more effective than the same treatments performed separately, i.e., using a unimodal treatment.

The results showed that patients who underwent a multimodal treatment had an improvement in performance regarding the knee-extensor muscle strength for both the affected and the unaffected lower limb. After stroke, people usually experience severe deconditioning depending on both functional sequelae and subsequent sedentary lifestyle. For these reasons, stroke rehabilitation, particularly the efforts of physical and occupational therapists, focuses on restoring impaired movement and associated functions. Since independence in walking has been correlated with lower-limb strength, muscle strength recovery represents a crucial aspect of rehabilitation [11,33]. In this regard, a growing number of studies suggest that strength training is a safe and effective intervention after stroke [34]. Therefore, although it is rarely practised in real-world settings, the implementation of high-intensity rehabilitation combining resistance and endurance training, as in multimodal treatment, should be imperative along the post-stroke rehabilitation pathway, aimed at minimising both the acute and long-term sequelae [35]. What has been found has an important clinical impact, as it firstly allows for the optimisation of rehabilitation outcomes. Secondly, it recognises intensity, in terms of dose and the type of exercise, as a relevant factor in poststroke recovery. Indeed, the Clinical Practice Guidelines (CPGs) from the American Physical Therapy Association (APTA) suggest, with a level A of evidence, that therapy targeted at improving motor functions should include repetitive and intense use of selective exercises tailored to a patient’s needs, which have an impact on the improvement in functional tasks and activities (e.g., standing up, static and dynamic balance, walking) [36]. Consistent with this suggestion, our results show that multimodal training improves the strength of knee extensors, and this could be beneficial for achieving an upright position and walking, thus allowing patients to improve independence levels in their ADLs. In fact, those tasks rely on valid activation and strength of lower limb extensor muscles [37]. As suggested by Severinsen, lower extremity muscle strength is related to walking performance, indicating the potential for endurance and resistance training in the rehabilitation of walking performance in chronic hemiparesis after stroke [38] and thus supporting current evidence with potential referral to specific rehabilitation programmes.

Our qualitative analysis of the included studies highlights that multimodal treatment is generally applied to patients with a time from stroke onset longer than 3 months. This is probably due to the need of clinical stability for providing high-intensity treatments.

The exercises mostly used in studies proposing multimodal approaches are the following: multi-district strength exercises (e.g., bodyweight or elastic bands exercises), breathing exercises, fast walking or cycling with a target heart rate zone to follow, ranging from 50% to 80% of maximum heart rate. In relation to the device used, the data suggested that the most commonly used rehabilitation device is the cycle ergometer, regardless of the manufacturer, thus demonstrating how technological devices can facilitate the delivery of multimodal therapy.

The meta-analyses conducted on all the remaining outcomes did not show statistically significant differences between unimodal and multimodal treatments, neither for the primary outcome (endurance) nor for secondary outcomes (i.e., walking speed and aerobic capacity). Heterogeneity was low for the endurance and walking outcomes, but high regarding the aerobic capacity outcome. This may depend on differences in the rehabilitation plan proposed in each trial included in the meta-analysis, as well as on their methodological quality. Indeed, the risk of bias assessment showed an important issue regarding “selection bias” in the reported results. The lack of good methodological reporting could have affected the outcome and, in turn, could have influenced the meta-analysis results.

The differences we found in the characteristics and dosage of rehabilitation plans are consistent with the present literature, as there is a lack of primary research studies adequately designed to answer the questions on characteristics and dosage that a multimodal treatment should consider to better influence the clinical outcomes. Even with regard to aerobic training, whose effect should be more related to improvements in aerobic capacity, walking speed, and endurance, the studies available in the literature report conflicting data and have not yet identified the optimal level of training intensity [39,40].

However, the results of this research reinforce the concept that the best post-stroke rehabilitation is always that which focuses on multiple goals simultaneously. This rehabilitation model is supported by previous studies, such as Megna’s study on the clinical efficacy of a combination of botulinum toxin type A and radial Extracorporeal Shock Wave Therapy [41].

### Study Limitations

This review has some limitations that need to be addressed. Firstly, we conducted each meta-analysis based on a few studies since the outcome measures considered by each study were not always the same, thus not allowing appropriate comparisons. Furthermore, for the studies included in the analysis, the differences in groups (e.g., age, inclusion or exclusion criteria, presence or absence of other pathologies) and intervention characteristics (e.g., type, duration, frequency) may have reduced the precision of our estimations.

## 5. Conclusions

Multimodal treatment is an emerging valid approach for the lower limb rehabilitation of stroke patients. In particular, treatments that combine resistance and endurance training have been shown to improve the recovery of knee-extensor muscle strength more than unimodal treatments.

Although there are still few studies focusing on this type of multimodal treatment approach in the field of lower limb rehabilitation after stroke, our analysis paves the way for its effective clinical application and, on the other hand, highlights the need for more primary research studies of good methodological quality. For an effective transfer of our findings to the clinic, it is essential to further study the multimodal treatment characteristics in terms of exercise load, training timings, and exercise delivery methods.

## Figures and Tables

**Table 1:** Characteristics of included studies.

First Author	Groups	N	Dose of Interventions	Description of the Multimodal Treatment	Description of the Unimodal Treatment	Outcome Measures	Conclusions
Bowden, M.G. (2020) [20]	(1) Multimodal group. (2) Unimodal group/usual care.	(1) 25. (2) 24.	(1) Total of 150.1 ± 15.8 min (two sessions each per week). (2) Total of144.3 ± 10.3 min.	Walking gait intensity: 110–125% of SSWS (self-selected walking speed), strength intensity: 75% of 1RM (repetition maximum), and a cardiovascular training HR (heart rate) target: ranging from 60% to 80% of the maximum HR.	Usual care, defined as the physical therapy treatment normally provided at each individual facility.	TUG test (Timed Up and Go), 10MWT (10-metre walking test), 2MWT (2-minute walk test), 5xSTS test (five times Sit to Stand), Tinetti (POMA), FIM (Functional Independence Measure)	Both the intervention and control groups improved significantly in each outcome measure, but the change scores from admission to discharge were consistently larger in the intervention group for all variables except the 5xSTS. An increased intervention intensity during the inpatient rehabilitation stay was a simple way to maximise patient function.
da Rosa Pinheiro, D.R. (2021) [21]	(1) Multimodal group. (2) Unimodal group/usual care.	(1) 10. (2) 10.	(1) One daily session (20′) of conventional physiotherapy + one daily session (20′) of cycle ergometer for 5 days. (2) Two daily sessions (20′) for 5 days.	One conventional physiotherapy session (5′ of stretch and strength exercises for biceps, triceps, quadriceps, hamstrings, gastrocnemius, 5′ of trunk control training and balance training, 5′ walking, and 5′ of breathing exercises) + one cycling session with an electric cycle ergometer (passive, active, and resistance exercises, with a biofeedback system for strength symmetry).	Conventional physiotherapy session (5′ of stretch and strength exercises for biceps, triceps, quadriceps, hamstrings, and gastrocnemius, 5′ of trunk control training and balance training, 5′ walking, and 5′ of breathing exercises).	Digital dynamometer (muscle strength), 10MWT, BBS (Berg Balance Scale), ICU Mobility Scale, Perme Score	Aerobic cycling training alongside conventional physiotherapy was effective in improving lower limb muscle strength, gait speed, balance, mobility and functionality.
Jin, H. (2012) [27]	(1) Multimodal group. (2) Unimodal group.	(1) 68. (2) 65.	(1) Five sessions (40′) per week for 8 weeks. (2) Five sessions (40′) per week for 8 weeks. Both of the two groups: balance (30′) and stretching (20′) exercises.	Aerobic cycling training with a target aerobic intensity of 50–70% HRR (heart rate reserve). Initial low intensity (40–50% HRR) for 5′ to 10′ increased 5′ every 2 weeks; intensity increased by 5% HRR every 2 weeks. Added 3% of body weight only for the paretic limb, 6′–10′ pedaled and 2′–3′ of rest.	Low-intensity (20–30% HRR) overground walking training.	Isokinetic dynamometer (knee muscle strength), 6MWT (6-minute walking test), peak VO2, BBS, modified Ashworth scale	The intensive aerobic cycling training with lower limb weights improve both cardiovascular fitness and walking ability, but the enhancements in cardiovascular fitness induced with training were not associated with the increases in walking capacity.
Lee, M.J. (2010) [23]	(1) Multimodal group. (2) Combinated (PRT + sham cycling). (3) Combinated (sham PRT + cycling). (4) Unimodal group/sham.	(1) 12. (2) 12. (3) 12. (4) 12.	(1) A total of 30 exercise sessions over a 10–12 weeks period with 3 sessions per week. (2) Same duration. (3) Same duration. (4) Same duration.	Progressive resistance training (PRT) consisting of two sets of eight repetitions at 50% of 1RM to start, then progressive to 80% + Aerobic cycle training consisting of 30′ of isokinetic leg cycling at 50% of VO2 peak to start, then progressive to 85%.	Sham PRT consisting of bilateral leg exercises using the same resistance of a training machine, but without any resistance other than the weight of the bar or gravity + sham cycling consisting of 30′ on motorised leg-passive cycling without any voluntary contraction.	Dynamometer (maximal force muscle), W (maximal muscle power), 1 TM, repetitions (muscle endurance)	Individuals who undertook PRT improved their muscle performance such as strength, peak power, and muscle endurance measures in both the affected and nonaffected lower limbs.
Lee, M.J. (2008) [24]	(1) Multimodal group. (2) Progressive resistance training. (3) Cycling. (4) Unimodal group/sham.	(1) 13. (2) 13. (3) 14. (4) 12.	(1) A total of 30 exercise sessions over a 10–12 weeks period with 3 sessions per week weeks. (2) Same duration. (3) Same duration. (4) Same duration.	Included 30′ cycling with motomed set at 40 rev/min and HR 50% of VO2 peak for 1–2 weeks, increased to 70% by week 4. After cycling, there is sham resistance training with two sets of eight repetitions for each exercise. PRT with pneumatic resistance equipment, two sets of eight repetitions unilaterally at 50% of baseline 1RM and progression to 80% by week 2.	Sham PRT consisting of bilateral leg exercises using the same resistance of a training machine but without any resistance other than the weight of the bar or gravity + sham cycling consisting of 30′ on motorised leg-passive cycling without any voluntary contraction.	Gait velocity, 6MWT, 10MWT, peak of HR and VO2, 1RM, dynamometer (muscle strength), SF-36 Questionnaire	Single-modality exercises targeted at existing impairments did not optimally address the functional deficits of walking but did ameliorate the underlying impairments. The underlying cardiovascular and musculoskeletal impairments were significantly modifiable years after stroke with targeted robust exercise.
Lee, Y.H. (2015) [25]	(1) Multimodal group. (2) Unimodal group/usual care.	(1) 14. (2) 12.	(1) A total of 1 h/day for 3 times/week for 16 weeks. (2) Unsystematic physical activities.	Each exercise intervention comprised a 5′ warm-up (standardised whole-body stretching, light walking, 10 stretching movement), a 30′ aerobic exercise (walking exercise, 10′ fast walking on a sloping way, 10′ walking in up-stairs), a 20′ resistance exercise (using elastic bands, lunges, squats, hip flexion/extension, hip abduction/adduction, knee flexion/extension, shoulder abduction/adduction, shoulder flexion/extension, and abdominal crunch/back extension), and a 5′ cool down (standardised whole-body stretching, light walking). AN exercise intensity target was established (60–70 HRR).	Unsystematic physical activities, no exercise intervention. They were asked to continue their normal daily activities.	TUG test, 6MWT, 10MWT, grip strength, CS30 test (30′′ Chair-Stand), CSR (Chair Sit and Reach), FRT (Functional Reach Test)	The combined aerobic and resistance exercise program significantly reduced central arterial stiffness and increased gait velocity in patients with chronic poststroke hemiparesis.
Marzolini, S. (2018) [22]	(1) Multimodal group. (2) Unimodal group.	(1) 33. (2) 35.	(1) Six months of aerobic training + resistance training (3 and 2 days/week, respectively). (2) Six months (five times/week).	Aerobic training (walking with stationary recumbent/upright cycling) + resistance training (multi-joint and single-joint exercises. One to two sets with 10/11 exercises: lunge, squat, abdominal curl-up, heel raise, bicep curl, supine triceps extension, affected-side hip flexion/extension, affected-side ankle dorsiflexion, single-limb knee extension, and flexion. Initially 50% or 60% 1RM then 70%).	Aerobic training (walking with stationary recumbent/upright cycling).	Sit-to-stand, 6MWT, stair climbing performance, VO2 peak, muscular strength	Despite the lack of advantage in 6MWT, combined training enhanced stroke recovery by improving components of cardiorespiratory fitness, muscular strength, and muscle mass accretion.
Son, S.M. (2014) [28]	(1) Multimodal group. (2) Unimodal group/usual care.	(1) 14. (2) 14.	(1) A total of 30 min per day, 5 days per week, for a period of 6 weeks. (2) Same duration.	Joint mobilization, muscle strengthening, balance training, resistance exercise training in a sitting position with a leg press (three sets—8 to 10 repetitions at 70% of 1RM).	Joint mobilization, muscle strengthening, and balance training.	BBS, TUG test, A-P (antero, posterior), M-L (medio, lateral) sway distances	Training involving muscle strength across multiple joints was an effective intervention for an improvement in the dynamic balance function of stroke patients.
Teixeira-Salmela, L.F. (1999) [26]	(1) Multimodal group. (2) Unimodal group/no treatment.	(1) 6. (2) 7.	(1) Three days a week for 10 weeks (exercise sessions lasted 60′–90′). (2) Ten weeks.	Each supervised training session included: 5′ to 10′ warm-up (calisthenics, mild exercises, ROM exercises), aerobic exercises (stepping or cycling with a HR 70% target), strength training, cool-down with relax, and strenght exercises.	No intervention.	Isokinetic peak, gait speed, stair climbing, HAP (Human Activity Profile), NHP (Nottingham Health Profile)	The 10-week combined program of muscle strengthening and physical conditioning resulted in gains in all measures of impairment and disability. These gains were not associated with measurable changes in spasticity in either the quadriceps or ankle plantarflexors.
Vahlberg, B. (2017) [29]	(1) Multimodal group. (2) Unimodal group/usual care.	(1) 20. (2) 23.	(1) Two times per week for 3 months. (2) Usual care.	PRB (Progressive Resistance Balance) training including 10′ warm-up (stationary cycling or walking), 45′ circuit class, and 20′ motivational session (discussions on issues and goals). Exercises followed HIFE (high-intensity functional exercise) program and consisted of lower limb strength and balance exercises, such as rising from a seated position and squats in parallel or walking stance or walking on a soft surface.	Usual care, individuals were encouraged to continue their regular activities.	PASE (Physical Activity Scale for the Elderly), 6MWT, BBS, SPPB (Short Physical Performance Battery), SPMSQ (Short Portable Mental Status Questionnaire), CRS (disease core risk), cholesterol HDL/LDL, BMI	Three-month progressive resistance and balance training was associated with reduced fat mass, which was related to improvements in walking capacity in older adults approximately one year after stroke.

## Data Availability

Data used to conduct this research are available upon request to the corresponding author.

## Supplementary Materials

The following supporting information can be downloaded at: https://www.mdpi.com/article/10.3390/healthcare12020189/s1, PRISMA checklist, Supplementary Materials A; Supplementary Materials B.
