# Supplementary material for: Multi-Modal versus Uni-Modal Treatment for the Recovery of Lower Limb Motor Function in Patients after Stroke: A Systematic Review with Meta-Analysis
Source: Healthcare (Basel). 2024 Jan 12;12(2):189. doi: 10.3390/healthcare12020189 (PMC10815740; doi:10.3390/healthcare12020189)
Supplement: Supplementary file 1 [file healthcare-12-00189-s001.zip › Supplementary A.docx]

**SUPPLEMENTARY MATERIALS A**

**Search strategy**

PUBMED (10/02/2022)

#1 "cerebrovascular disorders" [MeSH] OR "basal ganglia cerebrovascular disease" [MeSH] OR "brain ischemia" [MeSH] OR "intracranial hemorrhages" [MeSH] OR stroke [MeSH] OR "brain infarction" [MeSH] or "brain injuries" or "brain injury chronic" or stroke* OR poststroke OR post-stroke OR cerebrovasc* or "cerebral vascular" or cerebral OR "ischemi*"

#2 "multi-modal*" OR "exercise therapy" [Mesh] OR "aerobic exercise*" OR "exercise, physical" [Mesh] OR "Physical Therapy Modalities"[Mesh] OR "combined training" OR "Resistance Training"[Mesh] OR "Endurance Training"[Mesh] OR "endurance training" OR “resistance exercise*”

#3 "strength training" OR “strength” OR “strength exercise*”

#4 (#2 AND #3)

#5 “physical therapy” [MeSH Terms] OR “physical therapy” OR “conventional motor treatment*” OR “motor treatment*” OR “conventional motor therap*” OR “physiotherap*” OR physiotherapy OR kinesiotherapy OR “conventional train*” OR “motor rehabilitation” OR “usual care”

#6 "gait" [MeSH] OR gait OR walk* OR "lower limb function*" OR "lower limb impairment*" OR "Muscle Strength"[Mesh] OR "Muscle Strength" OR endurance OR resistan* OR "aerobic function*" OR "resistance" OR "lower limb muscle strength"

#7 (#1 AND #4 AND #5 AND #6)

COCHRANE (10/02/2022)

#1 MeSH descriptor: [Cerebrovascular Disorders] this term only

#2 MeSH descriptor: [Basal Ganglia Cerebrovascular Disease] explode all trees

#3 MeSH descriptor: [Brain Ischemia] explode all trees

#4 MeSH descriptor: [Intracranial Hemorrhages] explode all trees

#5 MeSH descriptor: [Stroke] explode all trees

#6 MeSH descriptor: [Brain Infarction] explode all trees

#7 (“brain injuries” or “brain injury chronic” or stroke* OR poststroke OR post-stroke OR cerebrovasc* or “cerebral vascular” or cerebral OR ischemi*)

#8 MeSH descriptor: [Exercise Therapy] this term only

#9 MeSH descriptor: [Exercise] this term only

#10 MeSH descriptor: [Physical Therapy Modalities] this term only

#11 MeSH descriptor: [Resistance Training] explode all trees

#12 MeSH descriptor: [Endurance Training] explode all trees

#13 ("multi-modal*" OR "aerobic exercise*" OR "combined training" OR "endurance training" OR “resistance exercise*”)

#14 ("strength training" OR “strength” OR “strength exercise*”)

#15 (“physical therapy” OR “conventional motor treatment*” OR “motor treatment*” OR “conventional motor therap*” OR “physiotherap*” OR physiotherapy OR kinesiotherapy OR “conventional train*” OR “motor rehabilitation” OR “usual care”)

#16 MeSH descriptor: [Gait] explode all trees

#17 MeSH descriptor: [Muscle Strength] explode all trees

#18 (gait OR walk* OR "lower limb function*" OR "lower limb impairment*" OR "Muscle Strength" OR endurance OR resistan* OR "aerobic function*" OR "resistance" OR "lower limb muscle strength")

#19 (#1 OR #2 OR #3 OR #4 OR #5 OR #6 OR #7)

#20 (#8 OR #9 OR #10 OR #11 OR #12 OR #13)

#21 (#14 AND #20)

#22 (#16 OR #17 OR #18)

#23 (#19 AND #21 AND #15 AND #22)

PEDro (10/02/2022)

#1 Stroke

#2 aerobic

#3 strength

#4 (#1 AND #2 AND #3)

EMBASE (10/02/2022)

1. ‘cerebrovascular disorders’/de OR ‘basal ganglia cerebrovascular disease’/de OR ‘brain ischemia’/de OR ‘intracranial hemorrhages’/de OR stroke/de OR ‘brain infarction’/de or ‘brain injuries’ or ‘brain injury chronic’ or stroke* OR poststroke OR post-stroke OR cerebrovasc* or ‘cerebral vascular’ or cerebral OR ischemi*

2. ‘multi-modal*’ OR ‘exercise therapy’/de OR ‘aerobic exercise*’ OR ‘exercise, physical’/de OR ‘Physical Therapy Modalities’/de OR ‘combined training’ OR ‘Resistance Training’/de OR ‘Endurance Training’/de OR ‘endurance training’ OR ‘resistance exercise*’

3. ‘strength training’ OR strength OR ‘strength exercise*’

4. (#2 AND #3)

5. ‘physical therapy’/de OR ‘physical therapy’ OR ‘conventional motor treatment*’ OR ‘motor treatment* OR ‘conventional motor therap*’ OR physiotherap* OR physiotherapy OR kinesiotherapy OR ‘conventional train*’ OR ‘motor rehabilitation’ OR ‘usual care’

6. ‘gait’/de OR gait OR walk* OR ‘lower limb function*’ OR ‘lower limb impairment*’ OR ‘Muscle Strength’/de OR ‘Muscle Strength’ OR endurance OR resistan* OR ‘aerobic function*’ OR ‘resistance’ OR ‘lower limb muscle strength’

7. (#1 AND #4 AND #5 AND #6)

SCOPUS (10/02/2022)

#1 (TITLE-ABS-KEY ("cerebrovascular disorders" OR "basal ganglia cerebrovascular disease" OR "brain ischemia" OR "intracranial hemorrhages" OR stroke OR "brain infarction" or "brain injuries" or "brain injury chronic" or stroke* OR poststroke OR post-stroke OR cerebrovasc* or "cerebral vascular" or cerebral OR ischemi*))

#2 (TITLE-ABS-KEY ("multi-modal*" OR "exercise therapy" OR "aerobic exercise*" OR "exercise, physical" OR "Physical Therapy Modalities" OR "combined training" OR "Resistance Training" OR "Endurance Training" OR "endurance training" OR “resistance exercise*”))

#3 (TITLE-ABS-KEY ("strength training" OR “strength” OR “strength exercise*”))

#4 (#2 AND #3)

#5 (TITLE-ABS-KEY (“physical therapy” OR “physical therapy” OR “conventional motor treatment*” OR “motor treatment*” OR “conventional motor therap*” OR “physiotherap*” OR physiotherapy OR kinesiotherapy OR “conventional train*” OR “motor rehabilitation” OR “usual care”))

#6 (TITLE-ABS-KEY ("gait" OR gait OR walk* OR "lower limb function*" OR "lower limb impairment*" OR "Muscle Strength" OR "Muscle Strength" OR endurance OR resistan* OR "aerobic function*" OR "resistance" OR "lower limb muscle strength"))

#7 (#1 AND #4 AND #5 AND #6)

WEB OF SCIENCE (10/02/2022)

#1 WC=(Rehabilitation)

#2 TS=("cerebrovascular disorders" OR "basal ganglia cerebrovascular disease" OR "brain ischemia" OR "intracranial hemorrhages" OR stroke OR "brain infarction" or "brain injuries" or "brain injury chronic" or stroke* OR poststroke OR post-stroke OR cerebrovasc* or "cerebral vascular" or cerebral OR ischemi*)

#3 TS=("multi-modal*" OR "exercise therapy" OR "aerobic exercise*" OR "exercise, physical" OR "Physical Therapy Modalities" OR "combined training" OR “Resistance Training" OR "Endurance Training" OR "endurance training" OR “resistance exercise*”)

#4 TS=(“strength training" OR “strength” OR “strength exercise*”)

#5 (#3 AND #4)

#6 TS=(“physical therapy” OR “physical therapy” OR “conventional motor treatment*” OR “motor treatment*” OR “conventional motor therap*” OR “physiotherap*” OR physiotherapy OR kinesiotherapy OR “conventional train*” OR “motor rehabilitation” OR “usual care”)

#7 TS=("gait" OR gait OR walk* OR "lower limb function*" OR "lower limb impairment*" OR "Muscle Strength" OR "Muscle Strength" OR endurance OR resistan* OR "aerobic function*" OR "resistance" OR "lower limb muscle strength")

#8 (#1 AND #2 AND #5 AND #6 AND #7)
