# Supplementary material for: Multi-Modal versus Uni-Modal Treatment for the Recovery of Lower Limb Motor Function in Patients after Stroke: A Systematic Review with Meta-Analysis
Source: Healthcare (Basel). 2024 Jan 12;12(2):189. doi: 10.3390/healthcare12020189 (PMC10815740; doi:10.3390/healthcare12020189)
Supplement: Supplementary file 1 [file healthcare-12-00189-s001.zip › Supplementary B.pdf]

Unique ID	Bowden MG 2020	Study ID	1	Assessor	AL, AM, LC
Ref or Label		Aim	assignment to intervention (the 'intention-to-treat' effect)		
Experimental		Comparator		Source	Journal article(s)
Outcome		Results		Weight	1

Domain	Signalling question	Response	Comments
Bias arising from the randomization process	1.1 Was the allocation sequence random?	N	Participants randomized according to clinical needs.
	1.2 Was the allocation sequence concealed until participants were enrolled and assigned to interventions?	N	
	1.3 Did baseline differences between intervention groups suggest a problem with the randomization process?	N	no significant differences at baseline for any variable.
	**Risk of bias judgement**	**High**	Participants randomized according to clinical needs; no significant differences at baseline for any variable.
Bias due to deviations from intended interventions	2.1.Were participants aware of their assigned intervention during the trial?	PY	No information provided, but probably patients and therapists were aware of the intervention received due to the nature of the intervention
	2.2.Were carers and people delivering the interventions aware of participants' assigned intervention during the trial?	PY	
	2.3. If Y/PY/NI to 2.1 or 2.2: Were there deviations from the intended intervention that arose because of the experimental context?	N	No deviations from intended interventions; participants balanced across groups
	2.4 If Y/PY to 2.3: Were these deviations likely to have affected the outcome?	NA	
	2.5. If Y/PY/NI to 2.4: Were these deviations from intended intervention balanced between groups?	NA	
	2.6 Was an appropriate analysis used to estimate the effect of assignment to intervention?	NI	No information
	2.7 If N/PN/NI to 2.6: Was there potential for a substantial impact (on the result) of the failure to analyse participants in the group to which they were randomized?	PY	28% of withdrawals
	**Risk of bias judgement**	**Some concerns**	No information provided about awareness of assignment, but probably patients and therapists were aware of the intervention received due to the nature of the intervention. No deviations from intended interventions; participants balanced across groups. No information about analysis to estimate the effect of assignment to intervention; 28% of withdrawals
Bias due to missing outcome data	3.1 Were data for this outcome available for all, or nearly all, participants randomized?	N	14 subjects withdrew.
	3.2 If N/PN/NI to 3.1: Is there evidence that result was not biased by missing outcome data?	N	No evidence
	3.3 If N/PN to 3.2: Could missingness in the outcome depend on its true value?	PY	Several patients dropped out because of transfers back to acute facilities. Three patients decreased in activity tolerance
	3.4 If Y/PY/NI to 3.3: Is it likely that missingness in the outcome depended on its true value?	PY	
	**Risk of bias judgement**	**High**	14 subjects withdrew. Several patients dropped out because of transfers back to acute facilities. Three patients decreased in activity tolerance
Bias in measurement of the outcome	4.1 Was the method of measuring the outcome inappropriate?	N	Appropriate outcome measures
	4.2 Could measurement or ascertainment of the outcome have differed between intervention groups?	N	Same assessment between groups
	4.3 Were outcome assessors aware of the intervention received by study participants?	Y	Outcome assessor not blinded
	4.4 If Y/PY/NI to 4.3: Could assessment of the outcome have been influenced by knowledge of intervention received?	PY	Some outcome measurements could have been influenced by the therapist who performed both treatments and assessments
	4.5 If Y/PY/NI to 4.4: Is it likely that assessment of the outcome was influenced by knowledge of intervention received?	PY	
	**Risk of bias judgement**	**High**	Appropriate outcome measures used; same assessment between groups. Outcome assessor was not blinded, thus some outcome measurements could have been influenced by the therapist who performed both treatments and assessments
Bias in selection of the reported result	5.1 Were the data that produced this result analysed in accordance with a pre-specified analysis plan that was finalized before unblinded outcome data were available for analysis?	NI	No information
	5.2 ... multiple eligible outcome measurements (e.g. scales, definitions, time points) within the outcome domain?	NI	No information
	5.3 ... multiple eligible analyses of the data?	NI	No information
	**Risk of bias judgement**	**Some concerns**	No protocol provided
**Overall bias**	**Risk of bias judgement**	**High**	The trial has many critic points that negatively influenced the methodological evaluation. It results with a high risk of bias, overall.

Unique ID	Da Rosa Pinheiro DR 2021	Study ID	2	Assessor	AL, AM, LC
Ref or Label		Aim	assignment to intervention (the 'intention-to-treat' effect)		
Experimental		Comparator		Source	Journal article(s)
Outcome		Results		Weight	1

Domain	Signalling question	Response	Comments
Bias arising from the randomization process	1.1 Was the allocation sequence random?	Y	The randomization was correctly performed, with an allocation ratio of 1:1 using opaque, sealed and sequentially numbered envelopes.
	1.2 Was the allocation sequence concealed until participants were enrolled and assigned to interventions?	Y	
	1.3 Did baseline differences between intervention groups suggest a problem with the randomization process?	N	No significant differences at baseline between groups.
	**Risk of bias judgement**	**Low**	The randomization was correctly performed, with an allocation ratio of 1:1 using opaque, sealed and sequentially numbered envelopes. No significant differences at baseline between groups.
Bias due to deviations from intended interventions	2.1.Were participants aware of their assigned intervention during the trial?	PY	Probably, patients, carers and therapists were aware of the assigned intervention, but this is due to the nature of it instead of to study quality.
	2.2.Were carers and people delivering the interventions aware of participants' assigned intervention during the trial?	PY	
	2.3. If Y/PY/NI to 2.1 or 2.2: Were there deviations from the intended intervention that arose because of the experimental context?	N	No deviation from intervention observed.
	2.4 If Y/PY to 2.3: Were these deviations likely to have affected the outcome?	NA	
	2.5. If Y/PY/NI to 2.4: Were these deviations from intended intervention balanced between groups?	NA	
	2.6 Was an appropriate analysis used to estimate the effect of assignment to intervention?	Y	All participants were analyzed.
	2.7 If N/PN/NI to 2.6: Was there potential for a substantial impact (on the result) of the failure to analyse participants in the group to which they were randomized?	NA	

	**Risk of bias judgement**	**Low**	Probably, patients, carers and therapists were aware of the assigned intervention, but this is due to the nature of it instead of to study quality.<br>No deviation from intervention observed.<br>All participants were analyzed.
**Bias due to missing outcome data**	3.1 Were data for this outcome available for all, or nearly all, participants randomized?	Y	All participants were analyzed.
	3.2 If N/PN/NI to 3.1: Is there evidence that result was not biased by missing outcome data?	NA	
	3.3 If N/PN to 3.2: Could missingness in the outcome depend on its true value?	NA	
	3.4 If Y/PY/NI to 3.3: Is it likely that missingness in the outcome depended on its true value?	NA	
	**Risk of bias judgement**	**Low**	All participants were analyzed.
**Bias in measurement of the outcome**	4.1 Was the method of measuring the outcome inappropriate?	N	Outcome measures were appropriate.
	4.2 Could measurement or ascertainment of the outcome have differed between intervention groups?	N	Same outcome measures between groups.
	4.3 Were outcome assessors aware of the intervention received by study participants?	PY	Health professionals have free access to the subjects, making it difficult to guarantee complete blinding of the evaluators.
	4.4 If Y/PY/NI to 4.3: Could assessment of the outcome have been influenced by knowledge of intervention received?	PY	
	4.5 If Y/PY/NI to 4.4: Is it likely that assessment of the outcome was influenced by knowledge of intervention received?	PN	
	**Risk of bias judgement**	**Some concerns**	Outcome measures were appropriate.<br>Same outcome measures between groups.<br>Health professionals have free access to the subjects, making it difficult to guarantee complete blinding of the evaluators
**Bias in selection of the reported result**	5.1 Were the data that produced this result analysed in accordance with a pre-specified analysis plan that was finalized before unblinded outcome data were available for analysis?	N	Reported results are not in accordance with the study protocol. In this paper, authors do not investigate all of the outcomes pre-specified.
	5.2 ... multiple eligible outcome measurements (e.g. scales, definitions, time points) within the outcome domain?	Y	The primary outcome specified in the study protocol is different from the one reported by authors in the paper.
	5.3 ... multiple eligible analyses of the data?	Y	
	**Risk of bias judgement**	**High**	Reported results are not in accordance with the study protocol. In this paper, authors do not investigate all of the outcomes pre-specified.<br>The primary outcome specified in the study protocol is different from the one reported by authors in the paper.
**Overall bias**	**Risk of bias judgement**	**High**	The randomization was correctly performed, with an allocation ratio of 1:1 using opaque, sealed and sequentially numbered envelopes.<br>No significant differences at baseline between groups.<br>Probably, patients, carers and therapists were aware of the assigned intervention, but this is due to the nature of it instead of to study quality.<br>No deviation from intervention observed.<br>All participants were analyzed.<br>All participants were analyzed.<br>Outcome measures were appropriate.<br>Same outcome measures between groups.<br>Health professionals have free access to the subjects, making it difficult to guarantee complete blinding of the evaluators<br>Reported results are not in accordance with the study protocol. In this paper, authors do not investigate all of the outcomes pre-specified.<br>The primary outcome specified in the study protocol is different from the one reported by authors in the paper.

**Unique ID**	Jin H 2012	**Study ID**	3	**Assessor**	AL, AM, LC
**Ref or Label**		**Aim**	assignment to intervention (the 'intention-to-treat' effect)		
**Experimental**		**Comparator**		**Source**	Journal article(s)
**Outcome**		**Results**		**Weight**	1

Domain	Signalling question	Response	Comments
**Bias arising from the randomization process**	1.1 Was the allocation sequence random?	NI	No information
	1.2 Was the allocation sequence concealed until participants were enrolled and assigned to interventions?	NI	
	1.3 Did baseline differences between intervention groups suggest a problem with the randomization process?	N	Groups were similar at baseline
	**Risk of bias judgement**	**Some concerns**	No information about randomization procedures; groups were similar at baseline
**Bias due to deviations from intended interventions**	2.1.Were participants aware of their assigned intervention during the trial?	PY	Probably, patients, carers and therapists were aware of the assigned intervention, but this is due to the nature of it instead of to study quality.
	2.2.Were carers and people delivering the interventions aware of participants' assigned intervention during the trial?	PY	
	2.3. If Y/PY/NI to 2.1 or 2.2: Were there deviations from the intended intervention that arose because of the experimental context?	N	There are no deviations from the intervention
	2.4 If Y/PY to 2.3: Were these deviations likely to have affected the outcome?	NA	
	2.5. If Y/PY/NI to 2.4: Were these deviations from intended intervention balanced between groups?	NA	
	2.6 Was an appropriate analysis used to estimate the effect of assignment to intervention?	Y	Absence of drop-outs
	2.7 If N/PN/NI to 2.6: Was there potential for a substantial impact (on the result) of the failure to analyse participants in the group to which they were randomized?	NA	
	**Risk of bias judgement**	**Low**	Probably, patients, carers and therapists were aware of the assigned intervention, but this is due to the nature of it instead of to study quality. There are no deviations from the intervention. Absence of drop-outs
**Bias due to missing outcome data**	3.1 Were data for this outcome available for all, or nearly all, participants randomized?	Y	All participants were analysed
	3.2 If N/PN/NI to 3.1: Is there evidence that result was not biased by missing outcome data?	NA	
	3.3 If N/PN to 3.2: Could missingness in the outcome depend on its true value?	NA	
	3.4 If Y/PY/NI to 3.3: Is it likely that missingness in the outcome depended on its true value?	NA	
	**Risk of bias judgement**	**Low**	All participants were analysed
**Bias in**	4.1 Was the method of measuring the outcome inappropriate?	N	Outcome measures were appropriate.
	4.2 Could measurement or ascertainment of the outcome have differed between intervention groups?	N	Same measurements between groups.
	4.3 Were outcome assessors aware of the intervention received by study participants?	PN	Authors defined the study as a "single-blind study", but there are no explicit information about awareness of assessors.

measurement of the outcome	4.4 If Y/PY/NI to 4.3: Could assessment of the outcome have been influenced by knowledge of intervention received?	NA	
	4.5 If Y/PY/NI to 4.4: Is it likely that assessment of the outcome was influenced by knowledge of intervention received?	NA	
	**Risk of bias judgement**	**Low**	Outcome measures were appropriate. Same measurements between groups. Authors defined the study as a "single-blind study", but there are no explicit information about awareness of assessors.
**Bias in selection of the reported result**	5.1 Were the data that produced this result analysed in accordance with a pre-specified analysis plan that was finalized before unblinded outcome data were available for analysis?	NI	No information
	5.2 ... multiple eligible outcome measurements (e.g. scales, definitions, time points) within the outcome domain?	NI	No information
	5.3 ... multiple eligible analyses of the data?	NI	No information
	**Risk of bias judgement**	**Some concerns**	No information provided.
**Overall bias**	**Risk of bias judgement**	**Some concerns**	The study is relatively well performed. However, some aspects related to the randomization process and to the selection of the reported results raise some concerns.

**Unique ID**	Lee MJ 2010	**Study ID**	4	**Assessor**	AL, AM, LC
**Ref or Label**		**Aim**	assignment to intervention (the 'intention-to-treat' effect)		
**Experimental**		**Comparator**		**Source**	Journal article(s)
**Outcome**		**Results**		**Weight**	1

Domain	Signalling question	Response	Comments
**Bias arising from the randomization process**	1.1 Was the allocation sequence random?	Y	Randomization and allocation methods were appropriate.
	1.2 Was the allocation sequence concealed until participants were enrolled and assigned to interventions?	Y	
	1.3 Did baseline differences between intervention groups suggest a problem with the randomization process?	N	No significant differences between groups at baseline.
	**Risk of bias judgement**	**Low**	Randomization and allocation methods were appropriate. No significant differences between groups at baseline.
**Bias due to deviations from intended interventions**	2.1.Were participants aware of their assigned intervention during the trial?	N	Some participants received real training and other received sham training. No information about awareness of carers and therapists of assigned intervention
	2.2.Were carers and people delivering the interventions aware of participants' assigned intervention during the trial?	NI	
	2.3. If Y/PY/NI to 2.1 or 2.2: Were there deviations from the intended intervention that arose because of the experimental context?	N	No deviation from intended intervention observed
	2.4 If Y/PY to 2.3: Were these deviations likely to have affected the outcome?	NA	
	2.5. If Y/PY/NI to 2.4: Were these deviations from intended intervention balanced between groups?	NA	
	2.6 Was an appropriate analysis used to estimate the effect of assignment to intervention?	N	4 participants not analyzed
	2.7 If N/PN/NI to 2.6: Was there potential for a substantial impact (on the result) of the failure to analyse participants in the group to which they were randomized?	N	The rate of participants lost to follow-up was low (8%)
	**Risk of bias judgement**	**Low**	Some participants received real training and other received sham training. No information about awareness of carers and therapists of assigned intervention. No deviation from intended intervention observed. 4 participants not analyzed, however, the rate of participants lost to follow-up was low (8%).
**Bias due to missing outcome data**	3.1 Were data for this outcome available for all, or nearly all, participants randomized?	Y	The rate of participants lost to follow-up was low (8%)
	3.2 If N/PN/NI to 3.1: Is there evidence that result was not biased by missing outcome data?	NA	
	3.3 If N/PN to 3.2: Could missingness in the outcome depend on its true value?	NA	
	3.4 If Y/PY/NI to 3.3: Is it likely that missingness in the outcome depended on its true value?	NA	
	**Risk of bias judgement**	**Low**	The rate of participants lost to follow-up was low (8%)
**Bias in measurement of the outcome**	4.1 Was the method of measuring the outcome inappropriate?	N	Outcome measures were appropriate
	4.2 Could measurement or ascertainment of the outcome have differed between intervention groups?	N	Same outcome measures between groups
	4.3 Were outcome assessors aware of the intervention received by study participants?	Y	Outcome assessor not blinded to intervention
	4.4 If Y/PY/NI to 4.3: Could assessment of the outcome have been influenced by knowledge of intervention received?	PY	Assessments were performed by the treating physical therapist. Furthermore, some outcome measures could be influenced by who performed the assessment.
	4.5 If Y/PY/NI to 4.4: Is it likely that assessment of the outcome was influenced by knowledge of intervention received?	PY	
	**Risk of bias judgement**	**High**	Outcome measures were appropriate. Same outcome measures between groups. Outcome assessor not blinded to intervention and some outcome measures could be influenced by who performed the assessment.
**Bias in selection of the reported result**	5.1 Were the data that produced this result analysed in accordance with a pre-specified analysis plan that was finalized before unblinded outcome data were available for analysis?	N	Reported results are not in accordance with the study protocol. In this paper, authors investigate only one of the outcome pre-specified (i.E. muscle performance).
	5.2 ... multiple eligible outcome measurements (e.g. scales, definitions, time points) within the outcome domain?	PN	Outcome measurements are in accordance with the study protocol
	5.3 ... multiple eligible analyses of the data?	PN	Data analysis are in accordance with the study protocol
	**Risk of bias judgement**	**Some concerns**	Reported results are not in accordance with the study protocol. In this paper, authors investigate only one of the outcome pre-specified (i.E. muscle performance). Outcome measurements and data analysis are in accordance with the study protocol
**Overall bias**	**Risk of bias judgement**	**High**	The outcome assessors were not blinded to intervention, resulting in a high risk of bias for this domain. Furthermore, discrepancies between study protocol and reported results were found, resulting in a high risk of bias, overall.

**Unique ID**	Lee MJ 2008	**Study ID**	5	**Assessor**	AL, AM, LC
**Ref or Label**		**Aim**	assignment to intervention (the 'intention-to-treat' effect)		
**Experimental**		**Comparator**		**Source**	Journal article(s)
**Outcome**		**Results**		**Weight**	1

Domain	Signalling question	Response	Comments

**Bias arising from the randomization process**	1.1 Was the allocation sequence random?	Y	Appropriate methods used to randomize and to allocate participants into groups
	1.2 Was the allocation sequence concealed until participants were enrolled and assigned to interventions?	Y	
	1.3 Did baseline differences between intervention groups suggest a problem with the randomization process?	N	No difference at baseline between groups
	**Risk of bias judgement**	**Low**	Appropriate methods used to randomize and to allocate participants into groups. No difference at baseline between groups
**Bias due to deviations from intended interventions**	2.1.Were participants aware of their assigned intervention during the trial?	PN	Patients received both in real and sham modalities. There are no sufficient informations about awarness of carers, but the awarness of the therapist is due to the nature of the treatment
	2.2.Were carers and people delivering the interventions aware of participants' assigned intervention during the trial?	PY	
	2.3. If Y/PY/NI to 2.1 or 2.2: Were there deviations from the intended intervention that arose because of the experimental context?	N	There are no deviations from the intervention
	2.4 If Y/PY to 2.3: Were these deviations likely to have affected the outcome?	NA	
	2.5. If Y/PY/NI to 2.4: Were these deviations from intended intervention balanced between groups?	NA	
	2.6 Was an appropriate analysis used to estimate the effect of assignment to intervention?	Y	ITT analysis performed at follow-up
	2.7 If N/PN/NI to 2.6: Was there potential for a substantial impact (on the result) of the failure to analyse participants in the group to which they were randomized?	NA	
	**Risk of bias judgement**	**Low**	Patients received both in real and sham modalities. There are no sufficient informations about awarness of carers, but the awarness of the therapist is due to the nature of the treatment. There are no deviations from the intervention and ITT analysis was performed at follow-up.
**Bias due to missing outcome data**	3.1 Were data for this outcome available for all, or nearly all, participants randomized?	PY	Following an intention-to-treat paradigm, any missing values at follow-up are brought forward from baseline data for the primary outcome (walking ability). No informations about other outcomes.
	3.2 If N/PN/NI to 3.1: Is there evidence that result was not biased by missing outcome data?	NA	
	3.3 If N/PN to 3.2: Could missingness in the outcome depend on its true value?	NA	
	3.4 If Y/PY/NI to 3.3: Is it likely that missingness in the outcome depended on its true value?	NA	
	**Risk of bias judgement**	**Low**	Following an intention-to-treat paradigm, any missing values at follow-up are brought forward from baseline data for the primary outcome (walking ability). No informations about other outcomes.
**Bias in measurement of the outcome**	4.1 Was the method of measuring the outcome inappropriate?	N	Outcome measures are appropriate.
	4.2 Could measurement or ascertainment of the outcome have differed between intervention groups?	N	No differences between groups.
	4.3 Were outcome assessors aware of the intervention received by study participants?	PY	All baseline testing are blinded. In the post-assessment, a single blinded observer assesses the primary outcome measures (e.g. walking ability). A single non-blinded assessor measured secondary outcomes (e.g. cardiorespiratory fitness, muscle strength, power, and endurance).
	4.4 If Y/PY/NI to 4.3: Could assessment of the outcome have been influenced by knowledge of intervention received?	PN	The assessment of the outcome could have been partially influenced, because the authors used objective measures.
	4.5 If Y/PY/NI to 4.4: Is it likely that assessment of the outcome was influenced by knowledge of intervention received?	NA	
	**Risk of bias judgement**	**Low**	Outcome measures are appropriate. No differences between groups. All baseline testing are blinded. In the post-assessment, a single blinded observer assesses the primary outcome measures (e.g. walking ability). A single non-blinded assessor measured secondary outcomes (e.g. cardiorespiratory fitness, muscle strength, power, and endurance). The assessment of the outcome could have been partially influenced, because the authors used objective measures.
**Bias in selection of the reported result**	5.1 Were the data that produced this result analysed in accordance with a pre-specified analysis plan that was finalized before unblinded outcome data were available for analysis?	Y	The data were in accordance with the protocol
	5.2 ... multiple eligible outcome measurements (e.g. scales, definitions, time points) within the outcome domain?	N	Data in accordance with the protocol
	5.3 ... multiple eligible analyses of the data?	N	Data in accordance with the protocol
	**Risk of bias judgement**	**Low**	The data were in accordance with the protocol
**Overall bias**	**Risk of bias judgement**	**Low**	The study is well performed and no risk of bias was identified.

**Unique ID**	Lee YH 2015	**Study ID**	6	**Assessor**	AL, AM, LC
**Ref or Label**		**Aim**	assignment to intervention (the 'intention-to-treat' effect)		
**Experimental**		**Comparator**		**Source**	Journal article(s)
**Outcome**		**Results**		**Weight**	1

Domain	Signalling question	Response	Comments
**Bias arising from the randomization process**	1.1 Was the allocation sequence random?	Y	Random and allocation methods appropriately performed.
	1.2 Was the allocation sequence concealed until participants were enrolled and assigned to interventions?	Y	
	1.3 Did baseline differences between intervention groups suggest a problem with the randomization process?	N	No baseline differences.
	**Risk of bias judgement**	**Low**	Random and allocation methods appropriately performed. No baseline differences
**Bias due to deviations from intended interventions**	2.1.Were participants aware of their assigned intervention during the trial?	PY	Probably participants, carers and therpapists were aware of the intervention received, but this is due to the nature of the intervention instead of to study quality
	2.2.Were carers and people delivering the interventions aware of participants' assigned intervention during the trial?	PY	
	2.3. If Y/PY/NI to 2.1 or 2.2: Were there deviations from the intended intervention that arose because of the experimental context?	N	No deviations from intervention observed
	2.4 If Y/PY to 2.3: Were these deviations likely to have affected the outcome?	NA	
	2.5. If Y/PY/NI to 2.4: Were these deviations from intended intervention balanced between groups?	NA	
	2.6 Was an appropriate analysis used to estimate the effect of assignment to intervention?	N	4 participants dropped out and they were not analyzed
	2.7 If N/PN/NI to 2.6: Was there potential for a substantial impact (on the result) of the failure to analyse participants in the group to which they were randomized?	Y	Drop-outs rate was high (13%)
	**Risk of bias judgement**	**High**	Probably participants, carers and therpapists were aware of the intervention received, but this is due to the nature of the intervention instead of to study quality. No deviations from intervention observed. 4 participants dropped out [illegible]
**Bias due to missing outcome data**	3.1 Were data for this outcome available for all, or nearly all, participants randomized?	N	13% of drop-outs
	3.2 If N/PN/NI to 3.1: Is there evidence that result was not biased by missing outcome data?	N	No evidence provided
	3.3 If N/PN to 3.2: Could missingness in the outcome depend on its true value?	PY	3 participants dropped out because they refused to participate [illegible]

data	3.4 If Y/PY/NI to 3.3: Is it likely that missingness in the outcome depended on its true value?	PY	3 participants dropped out because they refused to participate
	**Risk of bias judgement**	**High**	13% of drop-outs. No evidence provided about bias of missing data. 3 participants dropped out because they refused to participate
**Bias in measurement of the outcome**	4.1 Was the method of measuring the outcome inappropriate?	N	Outcome measures were appropriate.
	4.2 Could measurement or ascertainment of the outcome have differed between intervention groups?	N	Same outcome measures between groups.
	4.3 Were outcome assessors aware of the intervention received by study participants?	N	Outcome assessor was blinded
	4.4 If Y/PY/NI to 4.3: Could assessment of the outcome have been influenced by knowledge of intervention received?	NA	
	4.5 If Y/PY/NI to 4.4: Is it likely that assessment of the outcome was influenced by knowledge of intervention received?	NA	
	**Risk of bias judgement**	**Low**	Outcome measures were appropriate. Same outcome measures between groups.. Outcome assessor was blinded
**Bias in selection of the reported result**	5.1 Were the data that produced this result analysed in accordance with a pre-specified analysis plan that was finalized before unblinded outcome data were available for analysis?	NI	No information about study protocol provided
	5.2 ... multiple eligible outcome measurements (e.g. scales, definitions, time points) within the outcome domain?	NI	No information about study protocol provided
	5.3 ... multiple eligible analyses of the data?	NI	No information about study protocol provided
	**Risk of bias judgement**	**Some concerns**	No information about study protocol provided
**Overall bias**	**Risk of bias judgement**	**High**	The high drop-outs rate and the missingness of outcome data have a negative impact on the methodological quality of the study. Furthermore, no information about study protocol was provided.

**Unique ID**	Marzolini S 2018	**Study ID**	7	**Assessor**	AL, AM, LC
**Ref or Label**		**Aim**	assignment to intervention (the 'intention-to-treat' effect)		
**Experimental**		**Comparator**		**Source**	Journal article(s)
**Outcome**		**Results**		**Weight**	1

Domain	Signalling question	Response	Comments
**Bias arising from the randomization process**	1.1 Was the allocation sequence random?	Y	Randomization and allocation procedures were adequate
	1.2 Was the allocation sequence concealed until participants were enrolled and assigned to interventions?	Y	
	1.3 Did baseline differences between intervention groups suggest a problem with the randomization process?	PY	Baseline differences were adjusted through a post-hoc analysis
	**Risk of bias judgement**	**Some concerns**	Randomization and allocation procedures were adequate. Baseline differences were adjusted through a post-hoc analysis
**Bias due to deviations from intended interventions**	2.1.Were participants aware of their assigned intervention during the trial?	PY	Probably, participants, carers and therapists were aware of the assigned intervention, but this is due to the nature of it instead of to study quality
	2.2.Were carers and people delivering the interventions aware of participants' assigned intervention during the trial?	PY	
	2.3. If Y/PY/NI to 2.1 or 2.2: Were there deviations from the intended intervention that arose because of the experimental context?	N	No deviation from intervention observed
	2.4 If Y/PY to 2.3: Were these deviations likely to have affected the outcome?	NA	
	2.5. If Y/PY/NI to 2.4: Were these deviations from intended intervention balanced between groups?	NA	
	2.6 Was an appropriate analysis used to estimate the effect of assignment to intervention?	N	A total of 5 patients out of 73 were not analyzed
	2.7 If N/PN/NI to 2.6: Was there potential for a substantial impact (on the result) of the failure to analyse participants in the group to which they were randomized?	PN	The amount of dropped-out is similar in two groups. Totally, less than 10% of participants.
	**Risk of bias judgement**	**Low**	Probably, participants, carers and therapists were aware of the assigned intervention, but this is due to the nature of it instead of to study quality. No deviation from intervention observed. A total of 5 patients out of 73 were not analyzed. The amount of drop-outs is similar in two groups, totally, less than 10% of participants.
**Bias due to missing outcome data**	3.1 Were data for this outcome available for all, or nearly all, participants randomized?	N	93.2% of participants (68/73) completed the study. 2 drop-out in experimental group, 3 in control group.
	3.2 If N/PN/NI to 3.1: Is there evidence that result was not biased by missing outcome data?	N	No evidence provided
	3.3 If N/PN to 3.2: Could missingness in the outcome depend on its true value?	N	The reasons of drop-outs were not related to the intervention
	3.4 If Y/PY/NI to 3.3: Is it likely that missingness in the outcome depended on its true value?	NA	
	**Risk of bias judgement**	**Low**	93.2% of participants (68/73) completed the study. 2 drop-out in experimental group, 3 in control group.<br>No evidence provided about bias of missing outcome data. The reasons of drop-outs were not related to the intervention.
**Bias in measurement of the outcome**	4.1 Was the method of measuring the outcome inappropriate?	N	Outcome measures were appropriate.
	4.2 Could measurement or ascertainment of the outcome have differed between intervention groups?	Y	Some outcome measures were performed only to the intervention group.
	4.3 Were outcome assessors aware of the intervention received by study participants?	NA	
	4.4 If Y/PY/NI to 4.3: Could assessment of the outcome have been influenced by knowledge of intervention received?	NA	
	4.5 If Y/PY/NI to 4.4: Is it likely that assessment of the outcome was influenced by knowledge of intervention received?	NA	
	**Risk of bias judgement**	**High**	Outcome measures were appropriate. Some outcome measures were performed only to the intervention group.
**Bias in selection of the reported result**	5.1 Were the data that produced this result analysed in accordance with a pre-specified analysis plan that was finalized before unblinded outcome data were available for analysis?	NI	No information about study protocol
	5.2 ... multiple eligible outcome measurements (e.g. scales, definitions, time points) within the outcome domain?	NI	No information about study protocol
	5.3 ... multiple eligible analyses of the data?	NI	No information about study protocol
	**Risk of bias judgement**	**Some concerns**	No information about study protocol.
**Overall bias**	**Risk of bias judgement**	**High**	There are some critical aspects in the measurements of the outcome that could introduce a bias in the study results. Furthermore, some concerns arose for randomization procedures, and no information about study protocol were provided.

Unique ID	Son SM 2014	Study ID	8	Assessor	AL, AM, LC
Ref or Label		Aim	assignment to intervention (the 'intention-to-treat' effect)		
Experimental		Comparator		Source	Journal article(s)
Outcome		Results		Weight	1

Domain	Signalling question	Response	Comments
Bias arising from the randomization process	1.1 Was the allocation sequence random?	PY	Software was used to randomly allocate the subjects.
	1.2 Was the allocation sequence concealed until participants were enrolled and assigned to interventions?	PY	
	1.3 Did baseline differences between intervention groups suggest a problem with the randomization process?	N	No significant differences between groups at baseline.
	**Risk of bias judgement**	**Low**	Software was used to randomly allocate the subjects. No significant differences between groups at baseline.
Bias due to deviations from intended interventions	2.1.Were participants aware of their assigned intervention during the trial?	PY	Probably patients, carers and therapists were aware of the assigned intervention, but this is due to the nature of it instead of to study quality.
	2.2.Were carers and people delivering the interventions aware of participants' assigned intervention during the trial?	PY	
	2.3. If Y/PY/NI to 2.1 or 2.2: Were there deviations from the intended intervention that arose because of the experimental context?	N	No deviation from intervention observed
	2.4 If Y/PY to 2.3: Were these deviations likely to have affected the outcome?	NA	
	2.5. If Y/PY/NI to 2.4: Were these deviations from intended intervention balanced between groups?	NA	
	2.6 Was an appropriate analysis used to estimate the effect of assignment to intervention?	Y	All participants included in final analysis.
	2.7 If N/PN/NI to 2.6: Was there potential for a substantial impact (on the result) of the failure to analyse participants in the group to which they were randomized?	NA	
	**Risk of bias judgement**	**Low**	Probably patients, carers and therapists were aware of the assigned intervention, but this is due to the nature of it instead of to study quality. No deviation from intervention observed. All participants included in final analysis.
Bias due to missing outcome data	3.1 Were data for this outcome available for all, or nearly all, participants randomized?	Y	Outcome data available for all participants.
	3.2 If N/PN/NI to 3.1: Is there evidence that result was not biased by missing outcome data?	NA	
	3.3 If N/PN to 3.2: Could missingness in the outcome depend on its true value?	NA	
	3.4 If Y/PY/NI to 3.3: Is it likely that missingness in the outcome depended on its true value?	NA	
	**Risk of bias judgement**	**Low**	
Bias in measurement of the outcome	4.1 Was the method of measuring the outcome inappropriate?	N	Outcome measures were appropriate.
	4.2 Could measurement or ascertainment of the outcome have differed between intervention groups?	N	Same outcome measures between groups.
	4.3 Were outcome assessors aware of the intervention received by study participants?	NI	No specific information provided.
	4.4 If Y/PY/NI to 4.3: Could assessment of the outcome have been influenced by knowledge of intervention received?	PN	The researchers used a device (i.e. Good Balance system) to assess the dynamic balance, and clinical scales were repeated twice.
	4.5 If Y/PY/NI to 4.4: Is it likely that assessment of the outcome was influenced by knowledge of intervention received?	NA	
	**Risk of bias judgement**	**Low**	Outcome measures were appropriate. Same outcome measures between groups. No specific information provided about outcome assessors. The researchers used a device (i.e. Good Balance system) to assess the dynamic balance, and clinical scales were repeated twice.
Bias in selection of the reported result	5.1 Were the data that produced this result analysed in accordance with a pre-specified analysis plan that was finalized before unblinded outcome data were available for analysis?	NI	No information about study protocol.
	5.2 ... multiple eligible outcome measurements (e.g. scales, definitions, time points) within the outcome domain?	NI	No information about study protocol.
	5.3 ... multiple eligible analyses of the data?	NI	No information about study protocol.
	**Risk of bias judgement**	**Some concerns**	No information about study protocol.
Overall bias	**Risk of bias judgement**	**Some concerns**	The study is well performed, but the lack of information about study protocol raise some concerns about the selection of the reported results.

Unique ID	Teixeira-Salmela LF 1999	Study ID	9	Assessor	AL, AM, LC
Ref or Label		Aim	assignment to intervention (the 'intention-to-treat' effect)		
Experimental		Comparator		Source	Journal article(s)
Outcome		Results		Weight	1

Domain	Signalling question	Response	Comments
Bias arising from the randomization process	1.1 Was the allocation sequence random?	NI	No information about the method of randomization and allocation.
	1.2 Was the allocation sequence concealed until participants were enrolled and assigned to interventions?	NI	
	1.3 Did baseline differences between intervention groups suggest a problem with the randomization process?	PN	Baseline characteristics seem to be similar between groups.
	**Risk of bias judgement**	**Some concerns**	No information about the method of randomization and allocation. Baseline characteristics seem to be similar between groups.
Bias due to deviations from intended interventions	2.1.Were participants aware of their assigned intervention during the trial?	PY	Probably, participants, carers and therapists were aware of the assigned intervention, but this is due to the nature of it instead of to study quality.
	2.2.Were carers and people delivering the interventions aware of participants' assigned intervention during the trial?	PY	
	2.3. If Y/PY/NI to 2.1 or 2.2: Were there deviations from the intended intervention that arose because of the experimental context?	N	No deviation from intervention observed
	2.4 If Y/PY to 2.3: Were these deviations likely to have affected the outcome?	NA	
	2.5. If Y/PY/NI to 2.4: Were these deviations from intended intervention balanced between groups?	NA	
	2.6 Was an appropriate analysis used to estimate the effect of assignment to intervention?	Y	All participants were analyzed in their assigned group.
	2.7 If N/PN/NI to 2.6: Was there potential for a substantial impact (on the result) of the failure to analyse participants in the group to which they were randomized?	NA	

	**Risk of bias judgement**	**Low**	Probably, participants, carers and therapists were aware of the assigned intervention, but this is due to the nature of it instead of to study quality. No deviation from intervention observed. All participants were analyzed in their assigned group.
**Bias due to missing outcome data**	3.1 Were data for this outcome available for all, or nearly all, participants randomized?	Y	No drop-outs.
	3.2 If N/PN/NI to 3.1: Is there evidence that result was not biased by missing outcome data?	NA	
	3.3 If N/PN to 3.2: Could missingness in the outcome depend on its true value?	NA	
	3.4 If Y/PY/NI to 3.3: Is it likely that missingness in the outcome depended on its true value?	NA	
	**Risk of bias judgement**	**Low**	
**Bias in measurement of the outcome**	4.1 Was the method of measuring the outcome inappropriate?	N	Outcome measures were appropriate.
	4.2 Could measurement or ascertainment of the outcome have differed between intervention groups?	Y	Participants in the control group were assessed three times, whereas participants in the intervention group were assessed twice.
	4.3 Were outcome assessors aware of the intervention received by study participants?	NA	
	4.4 If Y/PY/NI to 4.3: Could assessment of the outcome have been influenced by knowledge of intervention received?	NA	
	4.5 If Y/PY/NI to 4.4: Is it likely that assessment of the outcome was influenced by knowledge of intervention received?	NA	
	**Risk of bias judgement**	**High**	Outcome measures were appropriate. Participants in the control group were assessed three times, whereas participants in the intervention group were assessed twice.
**Bias in selection of the reported result**	5.1 Were the data that produced this result analysed in accordance with a pre-specified analysis plan that was finalized before unblinded outcome data were available for analysis?	NI	No information about study protocol provided
	5.2 ... multiple eligible outcome measurements (e.g. scales, definitions, time points) within the outcome domain?	NI	No information about study protocol provided
	5.3 ... multiple eligible analyses of the data?	NI	No information about study protocol provided
	**Risk of bias judgement**	**Some concerns**	No information about study protocol provided.
**Overall bias**	**Risk of bias judgement**	**High**	Methodological aspects related to randomization process, measurement of the outcome and selection of the reported results raise a high risk of bias.

**Unique ID**	Vahlberg B 2017	**Study ID**	10	**Assessor**	AL, AM, LC
**Ref or Label**		**Aim**	assignment to intervention (the 'intention-to-treat' effect)		
**Experimental**		**Comparator**		**Source**	Journal article(s)
**Outcome**		**Results**		**Weight**	1

**Domain**	**Signalling question**	**Response**	**Comments**
**Bias arising from the randomization process**	1.1 Was the allocation sequence random?	Y	A computer-generated randomization process was implemented after the baseline assessment to ensure allocation concealment using simple randomization.
	1.2 Was the allocation sequence concealed until participants were enrolled and assigned to interventions?	Y	
	1.3 Did baseline differences between intervention groups suggest a problem with the randomization process?	N	No significant differences at baseline between groups.
	**Risk of bias judgement**	**Low**	A computer-generated randomization process was implemented after the baseline assessment to ensure allocation concealment using simple randomization. No significant differences at baseline between groups.
**Bias due to deviations from intended interventions**	2.1.Were participants aware of their assigned intervention during the trial?	PY	Probably, patients, carers and therapists were aware of the assigned intervention, but this is due to the nature of it instead of to study quality.
	2.2.Were carers and people delivering the interventions aware of participants' assigned intervention during the trial?	PY	
	2.3. If Y/PY/NI to 2.1 or 2.2: Were there deviations from the intended intervention that arose because of the experimental context?	N	No deviation from intervention observed
	2.4 If Y/PY to 2.3: Were these deviations likely to have affected the outcome?	NA	
	2.5. If Y/PY/NI to 2.4: Were these deviations from intended intervention balanced between groups?	NA	
	2.6 Was an appropriate analysis used to estimate the effect of assignment to intervention?	Y	All participants were analyzed.
	2.7 If N/PN/NI to 2.6: Was there potential for a substantial impact (on the result) of the failure to analyse participants in the group to which they were randomized?	NA	
	**Risk of bias judgement**	**Low**	Probably, patients, carers and therapists were aware of the assigned intervention, but this is due to the nature of it instead of to study quality. No deviation from intervention observed. All participants were analyzed.
**Bias due to missing outcome data**	3.1 Were data for this outcome available for all, or nearly all, participants randomized?	Y	All participants were analyzed.
	3.2 If N/PN/NI to 3.1: Is there evidence that result was not biased by missing outcome data?	NA	
	3.3 If N/PN to 3.2: Could missingness in the outcome depend on its true value?	NA	
	3.4 If Y/PY/NI to 3.3: Is it likely that missingness in the outcome depended on its true value?	NA	
	**Risk of bias judgement**	**Low**	
**Bias in measurement of the outcome**	4.1 Was the method of measuring the outcome inappropriate?	N	Outcome measures were appropriate.
	4.2 Could measurement or ascertainment of the outcome have differed between intervention groups?	N	Same outcome measures between groups.
	4.3 Were outcome assessors aware of the intervention received by study participants?	N	One independent assessor is blinded to participant allocation and conducts the measurements at the start of the intervention and at three months.
	4.4 If Y/PY/NI to 4.3: Could assessment of the outcome have been influenced by knowledge of intervention received?	NA	
	4.5 If Y/PY/NI to 4.4: Is it likely that assessment of the outcome was influenced by knowledge of intervention received?	NA	
	**Risk of bias judgement**	**Low**	Outcome measures were appropriate. Same outcome measures between groups. One independent assessor is blinded to participant allocation and conducts the measurements at the start of the intervention and at three months.
**Bias in selection of the reported result**	5.1 Were the data that produced this result analysed in accordance with a pre-specified analysis plan that was finalized before unblinded outcome data were available for analysis?	NI	No studies found with: NCT1161329
	5.2 ... multiple eligible outcome measurements (e.g. scales, definitions, time points) within the outcome domain?	NI	No studies found with: NCT1161329
	5.3 ... multiple eligible analyses of the data?	NI	No studies found with: NCT1161329

	**Risk of bias judgement**	**Some concerns**	No studies found with: NCT1161329
**Overall bias**	**Risk of bias judgement**	**Low**	The study is well performed, however, no studies were found with NCT1161329, there might be an error with the registration number.
